# Rational Combinations of Targeted Therapy and Immune Checkpoint Inhibitors in Head and Neck Cancers

**DOI:** 10.3389/fonc.2022.837835

**Published:** 2022-03-17

**Authors:** Annie Wai Yeeng Chai, Pei San Yee, Sok Ching Cheong

**Affiliations:** ^1^ Translational Cancer Biology Research Unit, Cancer Research Malaysia, Subang Jaya, Malaysia; ^2^ Department of Oral and Maxillofacial Clinical Sciences, Faculty of Dentistry, University of Malaya, Kuala Lumpur, Malaysia

**Keywords:** head and neck cancer, targeted therapy, immunotherapy, drug combinations, cancer genetics, immune checkpoint inhibitor (ICI)

## Abstract

Immunotherapy, especially the immune checkpoint inhibitors (ICIs) such as the pembrolizumab and nivolumab have contributed to significant improvements in treatment outcomes and survival of head and neck cancer (HNC) patients. Still, only a subset of patients benefits from ICIs and hence the race is on to identify combination therapies that could improve response rates. Increasingly, genetic alterations that occur within cancer cells have been shown to modulate the tumor microenvironment resulting in immune evasion, and these have led to the emergence of trials that rationalize a combination of targeted therapy with immunotherapy. In this review, we aim to provide an overview of the biological rationale and current strategies of combining targeted therapy with the approved ICIs in HNC. We summarize the ongoing combinatorial clinical trials and discuss emerging immunomodulatory targets. We also discuss the challenges and gaps that have yet to be addressed, as well as future perspectives in combining these different drug classes.

## Introduction

The immune checkpoint inhibitors (ICIs) have led to a paradigm shift in the treatment modalities for advanced-stage head and neck cancer (HNC) patients. In 2016, two ICIs targeting the programmed cell death -1 (PD-1), pembrolizumab and nivolumab were approved as second-line treatment for recurrent and metastatic (R/M) HNC (1, 2), and subsequently, pembrolizumab was approved as first-line treatment for advanced-stage HNC in 2019 (3). PD-1 is a membrane-bound receptor found on immune cells such as T cells, that upon binding to its ligand, programmed death ligand-1 (PD-L1) found on tumor cells, can prevent the attack by the cytolytic T cell, allowing cancer cells to escape from immune surveillance (4). Hence, ICIs that can block the PD1/PD-L1 interaction represents a promising therapeutic strategy (4).

Besides, being a relatively immune-inflamed cancer, the use of immunotherapy for HNC is rational since cytolytic T cells, and natural killer (NK) cells are present in high abundance in the tumor (5). However, HNC is also a relatively immunosuppressive tumor that have also developed other mechanisms to dampen or evade the immune system, suggesting that merely blocking these checkpoint molecules is not sufficient. These include tumor intrinsic genomic alterations that drive cancer development, presenting an opportunity to simultaneously suppress oncogenic signals and enhance immune activation to sensitize tumors to ICIs. Many molecular targeted agents in combination with ICIs are in clinical testing, and some have been approved as the standard of care, such as lenvatinib (6) and axitinib (7). In addition, actionable driver mutations such as in EGFR (8), BRAF (9), KRAS (10) could contribute to immune evasion, driving the emergence of new clinical trials to investigate the synergy between targeting these genetic events to improve ICIs response. Furthermore, common oncogenic mutations in HNC such as those leading to altered p53 or Wnt/beta-catenin signaling were found to modulate the immune microenvironment and potentially affect response towards ICIs (11–15). In particular, among metastatic HNC, p53 mutation was reported to be negative predictor of response towards ICIs (12).

The immunomodulatory mechanisms of the targeted agents might however be tissue- or context-specific (dependent on the genetic drivers). HNC comprises of tumors from the oral cavity, oropharynx, hypopharynx, and larynx. About 25% of HNC is estimated to be associated with human papilloma virus (HPV) infection, and this is especially prevalent and have prognostic implication in oropharyngeal cancer (16). Besides being molecularly and genetically distinct, HPV-positive HNC is also different from HPV-negative HNC in terms of its immune landscape (17), where HPV-positive HNC showed significantly higher immune cells infiltration and CD8 T cell activation (5). Although the KEYNOTE-012 trial has shown better response rates to pembrolizumab amongst HPV-positive HNC (18, 19), KEYNOTE-048 reported that clinical benefit is seen regardless of HPV status (20). More systematic review on the implications of HPV positivity in immunotherapy responses in HNC have been published recently in 17 and Wang et al., 2021 (21).

However, there remains a scarcity of information on the targeted agents that could synergize with ICIs in HNC. Hence, this review serves to provide an overview of the current strategies in combining targeted therapy with ICIs, as well as emerging immunomodulatory targets for HNC. Challenges and future perspectives will also be discussed.

## Targeted Agents Tested in Combination With ICIs in HNC

**Figure 1:**
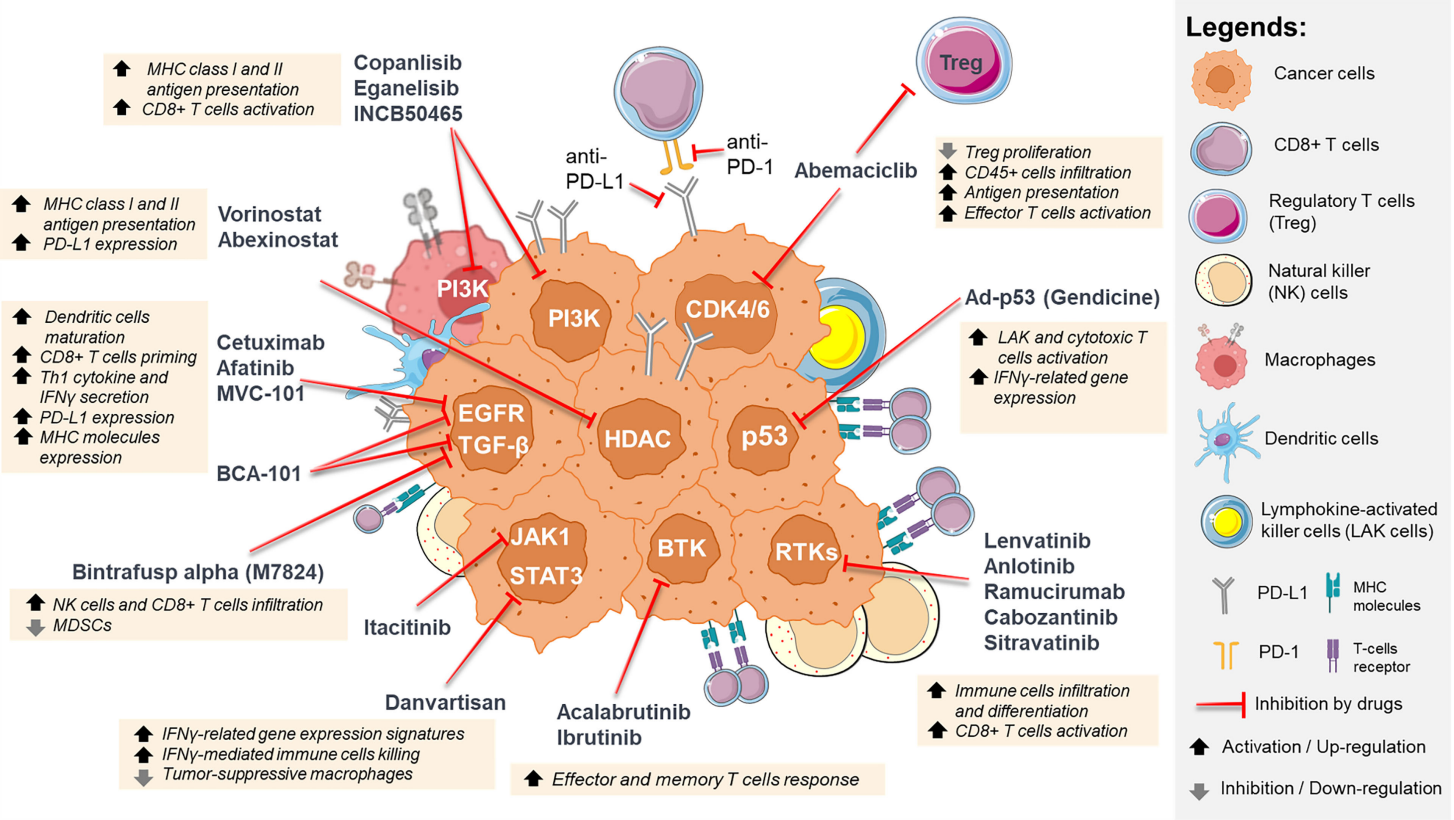
Summary of the immunomodulatory effects of inhibiting oncogenic signaling pathways in HNC. These targeted agents not only result in tumor-intrinsic killing, but also modulate the tumor microenvironment, such as increasing immune cell infiltration, activation and differentiation, increasing MHC class I and II antigen presentation, increasing PD-L1 expression, and inhibiting regulatory T cells proliferation. *For illustration purpose only, icons are not drawn in actual scale ratio. ** Some icons are taken from Servier Medical Art (smart.servier.com).

Clinical trials in HNC reporting combination therapies involving ICIs, were identified from clinicaltrials.gov, with the search terms of “Head and Neck Cancer” for “Condition or disease” and “pembrolizumab”, “nivolumab”, “atezolimumab”, or “durvalumab” under “Other terms” [Curated on 1^st^ Sep 2021]. A total of 49 trials involved the combination of molecular targeted agents with ICIs were summarized in **Table 1** (Details in **Supplementary Table 1** and **Supplementary Figure 1**). Whereas **Figure 1** provides a schematic summary of current strategies and their corresponding mechanisms of action in immunomodulation. Whilst most of these trials are still in their early phases, we hope to provide an overview and discuss the rationale of these combinations as detailed below.

**Table 1:** Overview of clinical trials involving the combination of targeted agents with ICIs for HNC patients.

Drug target	Targeted Drug Name	ICI	NCT number	Phase	Status	Biomarkers under investigation
EGFR	Cetuximab	Pembrolizumab	NCT03082534	Phase 2	Recruiting	PD-L1, EGFR expression, p16 status, EBV plasma DNA titres
		Nivolumab	NCT02764593	Phase 1	Active, not recruiting	–
		Nivolumab	NCT02124850	Phase 1	Terminated	NK activation, tumor infiltration, serum cytokines, mDC, T ell activation, tumor-antigen specific cytotoxic T lymphocyte induction
		Nivolumab	NCT03370276	Phase 1|Phase 2	Active, not recruiting	**-**
	Afatinib	Pembrolizumab	NCT03695510	Phase 2	Active, not recruiting	Unaltered MTAP level, EGFR amplification, PD-L1 expression
		Nivolumab	NCT03652233	Phase 1	Withdrawn	HPV status, somatic mutations in ERBB1, ERBB2, BRAF; expression levels of ErbB2 and PTEN, active CD8+ T cell density, expression and localization of PD-1, PD-L1, CTLA-4, TIM-3, LAG-3, OX40; circulating monocytic MDSCs, HBD3 expression
	MVC-101 (TAK-186)	Nivolumab	NCT04891718	Early Phase 1	Not yet recruiting	Cell death markers, T-cells , NK cells/myeloid cells, proinflammatory cytokines
EGFR and TGF-β	BCA-101	Pembrolizumab	NCT04429542	Phase 1	Recruiting	–
RTK	Lenvatinib (E7080/MK-7902)	Pembrolizumab	NCT04428151	Phase 2	Recruiting	–
		Pembrolizumab	NCT04199104	Phase 3	Recruiting	–
	Cabozantinib	Pembrolizumab	NCT03468218	Phase 2	Recruiting	**-**
		Nivolumab	NCT04514484	Phase 1	Recruiting	Immune checkpoint expression (PD-L1, B7x, HHLA2, B7H3), infiltrating immune cells level, tumor microenvironment biomarkers (VEGF, VEGFR, MET, AXL)
		Atezolizumab	NCT03170960	Phase 1|Phase 2	Recruiting	**-**
	Anlotinib	Pembrolizumab	NCT04999800	Phase 2	Recruiting	TMB, T cell gene expression
	Pexidartinib (PLX3397)	Pembrolizumab	NCT02452424	Phase 1|Phase 2	Terminated	–
	Ramucirumab (Cyramza)	Pembrolizumab	NCT03650764	Phase 1|Phase 2	Active, not recruiting	–
	Sitravatinib (MGCD516)	Nivolumab	NCT03575598	Early Phase 1	Completed	Tumor PD-L1 expression, density of immune cell in tumor and peripheral blood (T-cells, NK cells, myeloid-derived cells)
PI3K	Duvelisib	Pembrolizumab	NCT04193293	Phase 1|Phase 2	Suspended	–
	Copanlisib	Nivolumab	NCT03735628	Phase 1b|Phase 2	Active, not recruiting	–
	Eganelisib (IPI-549)	Nivolumab	NCT02637531	Phase 1	Active, not recruiting	–
JAK1 and PI3K-delta	Itacitinib (INCB039110) and INCB050465	Pembrolizumab	NCT02646748	Phase 1	Active, not recruiting	–
STAT3	Danvartirsan (AZD9150)	Durvalumab	NCT02499328	Phase 2	Active, not recruiting	PD-L1 expression
CDK4/6	Abemaciclib	Pembrolizumab	NCT03938337	Phase 2	Terminated	–
		Nivolumab	NCT03655444	Phase 1|Phase 2	Terminated	**-**
TGF-beta	Bintrafusp alfa (M7824 / MSB0011359C)	anti-PDL1	NCT02517398	Phase 1	Active, not recruiting	HPV status
		anti-PDL1	NCT03427411	Phase 2	Active, not recruiting	
BTK	Acalabrutinib (ACP-196)	Pembrolizumab	NCT02454179	Phase 2	Completed	–
	Ibrutinib	Nivolumab	NCT03646461	Phase 2	Recruiting	HPV status
HDAC	Abexinostat	Pembrolizumab	NCT03590054	Phase 1	Recruiting	–
	Vorinostat	Pembrolizumab	NCT04357873	Phase 2	Recruiting	–
		Pembrolizumab	NCT02538510	Phase 1|Phase 2	Active, not recruiting	PD-L1 expression, T cell phenotype, PD-1 family proteins expression
p53	Ad-p53	Pembrolizumab or Nivolumab	NCT02842125	Phase 1|Phase 2	Terminated	–
		Pembrolizumab/Nivolumab/Atezolizumab/Durvalumab	NCT03544723	Phase 2	Recruiting	–
Galectin-3	GR-MD-02	Pembrolizumab	NCT04987996	Phase 2	Suspended	GAL-3 expression, MDSC expression
		Pembrolizumab	NCT02575404	Phase 1	Active, not recruiting	CD4+ T cells, CD8+ T cells level
AURKA	Alisertib	Pembrolizumab	NCT04555837	Phase 1|Phase 2	Recruiting	T-cell repertoire, tumor infiltrating lymphocyte function
CXCR4	AMD3100	Pembrolizumab	NCT04058145	Phase 2	Withdrawn	–
SMO	Sonidegib	Pembrolizumab	NCT04007744	Phase 1	Recruiting	Immune cell markers, cytokines and soluble PD-L1 levels, level of Bcl-2 interacting mediator of cell death (BIM)
ATR	Elimusertib (BAY1895344)	Pembrolizumab	NCT04576091	Phase 1	Suspended	TMB, circulating Ki67+ CD8+ T cells
PDE5	Tadalafil	Pembrolizumab	NCT03993353	Phase 2	Recruiting	–
FUT8	SGN-2FF	Pembrolizumab	NCT02952989	Phase 1	Terminated	Fucosylation biomarker
PARP	Olaparib	Pembrolizumab	NCT04643379	Phase 2	Recruiting	–
EZH2	Tazemetostat	Pembrolizumab	NCT04624113	Phase 1|Phase 2	Recruiting	–
Arginase	INCB001158 (CB-1158)	Pembrolizumab	NCT02903914	Phase 1|Phase 2	Active, not recruiting	–
SYK/FLT3	TAK-659	Nivolumab	NCT02834247	Phase 1	Terminated	–
PPARα	TPST-1120	Nivolumab	NCT03829436	Phase 1	Recruiting	–
VEGF	Bevacizumab	Atezolizumab	NCT03818061	Phase 2	Active, not recruiting	HPV status, PD-1/PD-L1 expression, immune cells infiltration, cytokine production, microbiome
PTPN2	ABBV-CLS-579	anti-PD1	NCT04417465	Phase 1	Recruiting	Plasma/Serum metabolite M4 concentration
	ABBV-CLC-484	anti-PD1	NCT04777994	Phase 1	Recruiting	–

### EGFR Inhibitors

Epidermal growth factor receptor (EGFR) is overexpressed in more than 80% of HNC (22) and in addition to its oncogenic role, activated EGFR signaling modulates the immune microenvironment, helping tumors to escape from the immune system. Therefore, EGFR inhibitors have important immune-related mechanisms of action. Cetuximab, an IgG1 monoclonal antibody (mAb) against EGFR is the only approved targeted therapy for HNC. Cetuximab can exert its anti-tumor activity through antibody-dependent cell-mediated cytotoxicity that is triggered by the binding of the IgG-Fc part of cetuximab to the CD16 molecule on NK cells (23). Cetuximab triggers the maturation of dendritic cells (DC) causing the initiation of adaptive immune responses through CD8+ T cells priming and Th1 cytokine release, resulting in antigen-specific immune responses in HNC patients (24). EGFR signaling downregulates the antigen presentation machinery, enabling HNC to evade the immune system. Inhibiting EGFR signaling restores the expression of major histocompatibility complex (MHC) molecules, and enhances secretion of interferon-gamma (IFNγ) which increases PD-L1 expression on tumor and immune cells within the tumor microenvironment (TME) (25). Two Phase 2 trials are investigating the combination of cetuximab with either pembrolizumab [NCT03082534] or nivolumab [NCT03370276] whilst the combination of cetuximab and nivolumab is being tested in a Phase 1 trial for p16-negative local-regionally advanced HNC [NCT02764593]. In NCT03082534, where patients received cetuximab and pembrolizumab, a promising overall response rate (ORR) of 45% among platinum-refractory patients with no prior anti-EGFR/immunotherapy, was reported (26). On the other hand, for the nivolumab and cetuximab combination in platinum-refractory R/M HNC, the 1-year progression free survival (PFS) and overall survival (OS) rates were 19% and 44%, respectively. Although patients with no prior ICI showed improved PFS and OS compared to those with prior ICI, the difference was not significant (27).

High-throughput screening of small molecule inhibitors also showed that EGFR inhibitors (including erlotinib, afatinib and gefitinib) could enhance T cell-mediated killing (28). Clinically, afatinib is being tested with pembrolizumab for platinum-refractory R/M HNC in a Phase 2 trial [NCT03695510 – ALPHA study] unveiling an impressive ORR of 41.4% (29), a drastic increase compared to 16% or 10-10.8% for pembrolizumab and afatinib monotherapy respectively (30, 31). Biomarkers predictive of response were also investigated in this study, whereby *MTAP* loss or mutation was found to be associated with lower response rates, while *EGFR* amplification was associated with 100% ORR. On the other hand, high PD-L1 (CPS score ≥20) was associated with higher ORR (63% vs 35% in CPS score <20), albeit not statistically significant (Fisher’s exact p-value = 0.23) (29).

A Phase 1 study was initiated to study the pharmacodynamics of MVC-101 and nivolumab within the TME [NCT04891718]. MVC-101, is a conditionally active T cell engager that contains two anti-EGFR antibodies, designed for tumor cell- selective targeting (32). Similarly, BCA-101, a chimeric EGFR/TGFβ-targeting antibody is being tested in a Phase 1 trial with pembrolizumab [NCT04429542], which could address not only the immunosuppressive mechanisms (33), but also overcome the resistance to EGFR inhibitors brought on by upregulation of signaling (34, 35).

### RTK Inhibitors

Besides EGFR, other receptor tyrosine kinases (RTKs) implicated in HNC include the vascular endothelial growth factor receptor (VEGFR) and fibroblast growth factor receptor (FGFR). Clinical studies have demonstrated the synergistic effects of combining anti-angiogenic therapy with immunotherapy (36–38), as these could enhance immune cell differentiation and infiltration, and overcome the immune suppressive function of VEGF (38–40). In HNC, lenvatinib is being tested with pembrolizumab as first-line and second-line treatment [Phase 3, NCT04199104; Phase 2, NCT04428151]. Notably, lenvatinib has an immunomodulatory function by activating CD8+ T cells (41) and this combination treatment is approved for advanced RCC and endometrial cancer (42). Carbozantinib, an inhibitor of MET and VEGFR2 is also being tested with pembrolizumab [Phase 2, NCT03468218], nivolumab [Phase 1, NCT04514484] and atezolizumab [Phase 1/2, NCT03170960]. Further, anlotinib is being investigated with pembrolizumab as first-line therapy for R/M HNC with a combined positive score (CPS) ≥ 1 in a Phase 2 trial [NCT04999800]. Another VEGFR2 inhibitor ramucirumab, is undergoing Phase 2 trial with pembrolizumab [NCT03650764] whilst sitravatinib, a VEGFR inhibitor that also targets the TAM receptor family (TYRO3, AXL and MERTK) was combined with nivolumab in a window-of-opportunity (WOO) trial in neoadjuvant setting [NCT03575598] (42). Here, nine of ten patients showed clinical-to-pathological downstaging and immunophenotyping analyses confirmed the immunomodulatory effect of sitravatinib (42). Further investigation is needed to evaluate if this is contributed by the inhibition of the TAM receptors, which play a critical role in dampening NK cells mediated anti-tumor response (43, 44).

### PI3K Inhibitors

Oncogenic mutations in *PIK3CA* and dysregulation of PI3K signaling have been actively investigated as a therapeutic target in HNC (45, 46). Whilst exerting tumor intrinsic effect on HNC growth, targeting PI3K signaling could also reverse its immune-suppressive effects. Activating mutations in *PIK3CA* or *PTEN* loss which are both frequent events in HNC, represses the induction of MHC class I and II expression by IFNγ (47). PI3K inhibitors, dactolisib and pictilisib can induce MHC molecules expression in HNC cell lines (47). PD-1 blockade has been shown to upregulate another checkpoint receptor, Tim-3 *via* AKT/S6 pathway which could allow the escape of anti-PD-1 blockade in the TME, providing further support for concomitant targeting AKT/S6 to improve the efficacy of ICIs (48). Recently, targeting of PI3Kγ/δ in the leucocytes using TG100-115 or IPI-145 has been shown to improve ICIs efficacy in pre-clinical HNC models (27–29). This is likely attributed by the abrogation of myeloid-derived suppressor cells (MDSC)-mediated immune suppression (49). However, at high dose, IPI-145 was shown to negatively impact the priming and effector function of antigen-specific T cells, reversing the tumor control achievable by low dose IPI-145 (49). Considering the narrow therapeutic window in the combination of PI3K inhibitors with ICI, careful trial design in scheduling and dosing is warranted.

Currently, three PI3K inhibitors are being tested with ICIs in HNC trials. Previous pharmacodynamic study of copanlisib [NCT02155582] showed suppression of factors associated with macrophages and regulatory T cells (Tregs), supporting the rationale for the combination with nivolumab [NCT03735628]. Another Phase 1 trial combined eganelisib (PI3Kγ inhibitor) with nivolumab, demonstrating positive response (ORR=20%) and disease control rates of 40% at interim analysis (50), among those who had one or two prior lines of chemotherapy. Following the encouraging outcome, a Phase 2 WOO study [NCT03795610] is ongoing for locally advanced HNC to evaluate the effect of eganelisib in modulating a PI3Kγ-mediated immune-suppressive signature. Interim biomarker analysis from another Phase 1 dose-finding trial [NCT02646748] showed that the PI3K-δ inhibitor (INCB050465) enhanced T cell activation, leading to the improved outcome when combined with pembrolizumab (51).

### JAK/STAT Inhibitors

Activated JAK/STAT signaling increases tumor cell proliferation, treatment resistance and immune evasion in HNC (52, 53). A Phase 1 trial [NCT02646748] investigated itacitinib, a JAK inhibitor with pembrolizumab, but was inferior to the arm with PI3K-δ inhibitor plus pembrolizumab (51). The antagonistic effect of JAK inhibition with pembrolizumab, may be caused by the suppression of STAT1, which impairs IFNγ-mediated immune cell killing (54, 55). Indeed, trials in other solid tumors were terminated early due to poor efficacy, speculated to be caused by the impairment of the immune cell function with JAK inhibition (53, 56). While the inhibition of JAK has yielded contradicting results (53, 56), the selective inhibition of STAT3 is more promising as this targets both tumor intrinsic pathway signaling as well as STAT3 inhibition within the immune cells (54). Recently, the development of therapeutic antisense oligonucleotide such as danvartisan (AZD5190) has enabled the selective targeting of STAT3. On-treatment biopsies analysis from a Phase 1 study [NCT01563302] showed that danvatirsan upregulated IFNγ-related gene expression signatures (GES) (54). Furthermore, danvatirsan promoted pro-inflammatory cytokine expression and depleted immunosuppressive macrophages, enhancing T cell killing in syngeneic models (54). A Phase 2 trial investigating danvatirsan plus durvalumab (anti-PD-L1) as second-line treatment, is currently ongoing [NCT02499328]. Interim analysis showed an enhanced response (ORR of 26%) among second-line anti-PD-L1 naïve patients, compared with durvalumab with a CXCR2 inhibitor (AZD5069; ORR of 10%) with responses independent of HPV or PD-L1 status (57), and with on-treatment biopsies showing upregulation of IFNγ GES (57).

### Cell Cycle Inhibitors

The cell cycle pathway is altered in almost all HNC (58), with CDK4 and CDK6 among the most studied drug targets (59). Abemaciclib, an inhibitor of CDK4/6 showed anti-tumor effect in HNC patient-derived xenograft harboring CCND1 amplification and/or CDKN2A mutation. The immune-modulatory functions of CDK4/6 inhibitors include increasing antigen representation, promoting infiltration of CD45+ cells into the tumor, activating effector T cells and inhibiting Treg proliferation (60, 61). A WOO trial [NCT04169074] is accessing the immune-modulation function of abemaciclib, among HPV-negative HNC patients in a neoadjuvant setting. Disappointingly, two trials in HNC [NCT03938337, NCT03655444] which tested the combination of abemaciclib with pembrolizumab and nivolumab respectively, were terminated early. The former was terminated due to high incidence of pulmonary toxicities, including death. Consistently, adverse events were also reported for non-small-cell lung cancer (62). Given the poor risk-benefit profile of the combination from several trials, the combination of CDK4/6 inhibitors with ICI would require dose adjustments and biomarkers that could identify the most suitable patients for this treatment.

### TGF-β Inhibitors

Elevated expression of TGF-β is common in HNC and is associated with advanced disease and poor clinical outcome (63). Importantly, TGF-β signaling regulates both innate and adaptive immune signaling, impacting diverse types of immune cells (64). A first-in-class, bifunctional fusion protein, bintrafusp alfa (M7824) has been developed, composing of the extracellular domain of the human TGF-β receptor II, fused with an IgG1 avelumab-like anti-PDL1 antibody (65). M7824 increased infiltration of CD8+ T cells and NK cells into the tumor, while depleting MDSC in a murine model (66). Results from a Phase 1 study [NCT02517398] showed an ORR of 13%-16%, and a total clinical response rate of 22% (65). Encouragingly, early-phase trials of M7824 demonstrated efficacy seen in heavily pre-treated patients with “cold” tumors, whom tumor progressed/recurred after platinum therapy and are immunotherapy naive (66). Responses were seen regardless of PD-L1 status (ORR of 12% in PD-L1-positive vs 17% in PD-L1-negative), but HPV-positive HNC had significantly higher response rate (ORR of 33% in HPV-positive versus 5% in HPV-negative), perhaps due to the more enriched pre-existing immune response in these viral-driven cancers (65). As a result, a Phase 2 trial is now ongoing for HPV-associated oropharyngeal cancers [NCT03427411].

### Others

Bruton tyrosine kinase (BTK) has recently shown ectopic expression in some solid tumors (67). Despite the low efficacy as a single agent, preclinical studies showed synergy in improving survival when BTK inhibitors were combined with immunotherapy (67), demonstrating enhanced effector and memory T cells responses (68). Ibrutinib with nivolumab are being tested in a Phase 2 trial for R/M HNC [NCT03646461], with patients stratified by HPV status and randomized in a 1:1 ratio to ibrutinib + cetuximab or ibrutinib + nivolumab arms. Another BTK inhibitor, acalabrutinib is being tested with pembrolizumab in advanced HNC patients, unfortunately all patients have discontinued treatment due to the lack of efficacy (ORR for combination = 16.7% vs pembrolizumab = 18.9%) and increase in adverse events [NCT02454179]. The limited clinical benefit and increased toxicity make it unfavorable to pursue BTK inhibitor in combination with ICI in HNC.

HNC shows hypoacetylated chromatin and inhibition of the histone deacetylases (HDAC) is seen as a promising strategy in targeting the cancer-stem-cell population of HNC (69). HDAC inhibitors can upregulate PD-L1 and MHC class I/II expression (70) and are being actively investigated for their role in potentiating response to ICIs (71). A recently completed Phase 2 trial [NCT02538510] showed favorable activity of vorinostat and pembrolizumab in HNC, with a higher ORR of 32% than pembrolizumab alone, albeit at the expense of higher toxicity (72). Another two trials combining either vorinostat [NCT04357873] or abexinostat [NCT03590054] with pembrolizumab are in Phase 1.

Gene therapy that restores functional tumor suppressor has also been explored. Gendicine, a gene therapy to deliver functional wildtype p53 into tumor cells was approved for HNC in 2003 in China (73). Experimental data showed that gendicine can activate lymphokine-activated killer cells (LAK) and cytotoxic T cells (73). A Phase 2 trial [NCT03544723] is ongoing to investigate the combination of gendicine with clinicians’ choice of ICIs. Preliminary gene expression analysis comparing pre- and post- gendicine-treated HNC tumor revealed upregulation of IFNγ-signaling genes, decreased TGF-β and β-catenin signaling (74) and increased CD8+ T cells signature, which is associated with increased clinical responses to ICIs (74).

New immunomodulatory targets have also been identified *in vivo* immuno-CRISPR/Cas9 screens and *PTPN2* is among one of the top hits (75). CRISPR-mediated knockout of PTPN2 sensitized response towards anti-PD1 treatment, by enhancing IFNγ-mediated antigen presentation and increasing cytotoxic Tim-3+ CD8+ Tcells (76, 77). Two novel inhibitors targeting *PTPN2* - BBV-CLS-579 [NCT04417465] and ABBV-CLS-484 [NCT04777994] are now being tested in combination with anti-PD1 in Phase 1 clinical trials, for LA and metastatic solid tumors, including HNC. Other targets from these screens shown to be able to enhance immune response through T cell suppression, M1 macrophage polarization and/or PD-L1 regulation include *Asf1a (* 78 *)* and *eIF5B (* 79 *)*.

Other drugs targeting Galectin-3 (GR-MD-02), AURKA (alisertib), SMO (sonidegib), PDE5 (tadalafil), PARP (olaparib), EZH2 (tazemotostat), arginase (INCB001158) are in Phase 1 or 2 trials, in combination with pembrolizumab (**Table 1**). The PPARα antagonist, TPST-1120 in combination with nivolumab is in Phase 1 for advanced HNC [NCT03829436]. Whereas bevacizumab, a mAb targeting soluble VEGF is being tested with atezolizumab in a Phase 1 trial [NCT03818061]. Results from these trials will provide further insights into pathways that confer immunomodulatory effects in HNC.

## Emerging Immunomodulatory Targets

Other emerging immunomodulatory targets in HNC preclinical studies or in clinical studies of other cancers are discussed below.

### MAPK Pathway

The mitogen-activated protein kinases (MAPKs) are key regulators of cellular proliferation contributing to HNC progression (80). Apart from the established oncogenic roles, MAPK signaling pathway also promotes immunosuppression. In HNC cell lines, trametinib could enhance expressions of MHC class I and PD-L1 *via* STAT3 activation (81). The combination of trametinib with anti-PD-L1 significantly delayed tumor growth in HNC syngeneic models, possibly *via* increased infiltration of CD8+ T cells and enhanced antigen-presentation (81). Interestingly, HRAS mutations which are predominantly found in HNC are associated with elevated antitumor immune signatures (11). MAPK-mutated HNC wasa also found to be CD8+ T cell inflamed and inherently harbor immunoactive TME (82). Consistently, HNC with MAPK pathway mutations has been associated with better patient survival upon anti-PD1/PD-L1 treatment (82). Although no combinatorial trials for HNC are being tested currently, findings using syngenic models demonstrated that preceding MAPK inhibition with anti-PD-1/L1 could enhance CD8+ T cells clonal expansion and clinical investigation is warranted (83).

### MTOR Pathway

Mammalian target of Rapamycin (mTOR) is one of the critical intracellular kinases within the oncogenic PI3K/AKT pathway. A combination of rapamycin and anti-PD-L1 mAb increased the durability of tumor responses and survival in highly immunogenic 4MOSC1 tumors (84). The addition of rapamycin preserved antigen-specific CD8+ tumor-infiltrating lymphocytes (TIL) and enhanced IFNγ secretion in both peripheral and infiltrating CD8+ T cells, leading to upregulation of MHC class I expression (84). Whilst it appears counterintuitive to use rapamycin which is clinically used as an immunosuppressant in transplant patients, emerging evidence has shown that inhibiting mTOR confers immune-activating function, particularly by promoting CD8+ T cells generation (85), suggesting therapeutic opportunities to boost the efficacy of immunotherapy.

## Discussion

### Challenges and Future Perspective

The scarcity of suitable mouse models for the immunological study of HNC is a major challenge. Only lately, the 4−nitroquinoline−1−oxide induced tumor in the immunocompetent C57BL/6 mice has led to the establishment of the JC1-2 and 4-MOSC syngeneic models (86, 87). These models are instrumental in identifying novel immunomodulatory targets and evaluating new immune-oncology drugs for HNC. The dynamic changes in the TME and immune landscape during treatment can be comprehensively studied using high-resolution technologies such as the single-cell sequencing and spatial-omics, affording the opportunity to identify immune-modulatory effects of specific drug and drug combinations. Intratumor heterogeneity and plasticity of the TME are two other challenges that could influence the outcome of combining targeted therapy with immunotherapy. Further, an appropriate dosage, the timing and sequence of the combination needs to be considered carefully for optimal benefit-risk profile.

Finally, the successful application of precision medicine is highly reliant on clinically-relevant predictive biomarkers, a major challenge that is still being overcome. In HNC, other than PD-L1 CPS for pembrolizumab, no other clinical biomarker has made its way to the clinic. The advent of genomic, transcriptomic, proteomic, and immunomic, which allow comprehensive integrative analysis, could lead to a better understanding of clinical response, driving the identification of specific biomarkers for each drug combination.

## Conclusion

Combinations of ICIs with targeted agents have shown promising clinical efficacy, particularly those targeting EGFR (cetuximab, afatinib), RTK (lenvatinib, sitravatinib), STAT3 (danvartisan) and TGF-β (M7824). As most trials are still in early stages, an improved understanding of the genomics and immunology interplay and the lessons learned from trials in other solid tumors could guide rational and optimal trial designs in HNC. Furthermore, the increasing availability of HNC syngenic models and the application of advanced omic technologies could further fuel the development of combinatorial targeted therapy with ICIs further maximizing the clinical benefit of immunotherapy.

## Author Contributions

Conceptualization - AC and SC. Data curation, figure preparation - AC. Writing, reviewing & editing - AC, PY, and SC. Supervision – SC. All authors contributed to the article and approved the submitted version.

## Conflict of Interest

The authors declare that the research was conducted in the absence of any commercial or financial relationships that could be construed as a potential conflict of interest.

## Publisher’s Note

All claims expressed in this article are solely those of the authors and do not necessarily represent those of their affiliated organizations, or those of the publisher, the editors and the reviewers. Any product that may be evaluated in this article, or claim that may be made by its manufacturer, is not guaranteed or endorsed by the publisher.
