# Supplementary material for: Rational Combinations of Targeted Therapy and Immune Checkpoint Inhibitors in Head and Neck Cancers
Source: Front Oncol. 2022 Mar 17;12:837835. doi: 10.3389/fonc.2022.837835 (PMC8968950; doi:10.3389/fonc.2022.837835)
Supplement: Supplementary file 1 [file Presentation_1.pptx]

## Slide 1
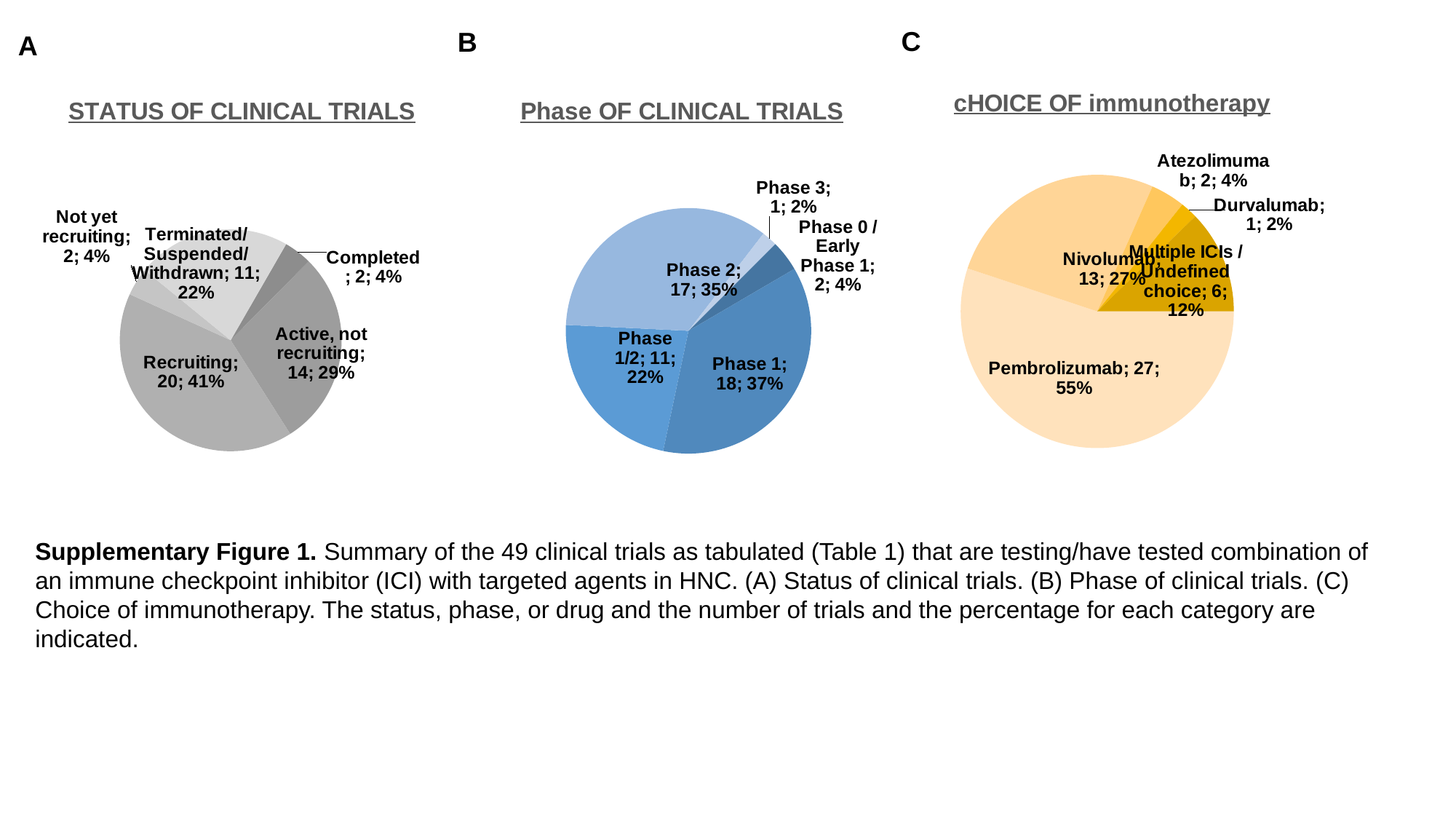

C
B
A
### Chart: cHOICE OF immunotherapy
| Category | |
|---|---|
| Pembrolizumab | 27.0 |
| Nivolumab | 13.0 |
| Atezolimumab | 2.0 |
| Durvalumab | 1.0 |
| Multiple ICIs / Undefined choice | 6.0 |
### Chart: Phase OF CLINICAL TRIALS
| Category | Number of trials |
|---|---|
| Phase 0 / Early Phase 1 | 2.0 |
| Phase 1 | 18.0 |
| Phase 1/2 | 11.0 |
| Phase 2 | 17.0 |
| Phase 3 | 1.0 |
### Chart: STATUS OF CLINICAL TRIALS
| Category | Number of trials |
|---|---|
| Status of trial | None |
| Completed | 2.0 |
| Active, not recruiting | 14.0 |
| Recruiting | 20.0 |
| Not yet recruiting | 2.0 |
| Terminated/Suspended/Withdrawn | 11.0 |Supplementary Figure 1. Summary of the 49 clinical trials as tabulated (Table 1) that are testing/have tested combination of an immune checkpoint inhibitor (ICI) with targeted agents in HNC. (A) Status of clinical trials. (B) Phase of clinical trials. (C) Choice of immunotherapy. The status, phase, or drug and the number of trials and the percentage for each category are indicated.
